# Heterogeneous integration of high-k complex-oxide gate dielectrics on wide band-gap high-electron-mobility transistors

**DOI:** 10.1038/s44172-024-00161-z

**Published:** 2024-01-19

**Authors:** Jongho Ji, Jeong Yong Yang, Sangho Lee, Seokgi Kim, Min Jae Yeom, Gyuhyung Lee, Heechang Shin, Sang-Hoon Bae, Jong-Hyun Ahn, Sungkyu Kim, Jeehwan Kim, Geonwook Yoo, Hyun S. Kum

**Affiliations:** 1 Department of Electrical and Electronic Engineering, Yonsei University, Seoul, South Korea; 2 Department of Electronic Engineering, Soongsil University, Seoul, South Korea; 3 Department of Mechanical Engineering, Massachusetts Institute of Technology, Cambridge, MA USA; 4 Research Laboratory of Electronics, Massachusetts Institute of Technology, Cambridge, MA USA; 5 Department of Nanotechnology and Advanced Materials Engineering, Sejong University, Seoul, South Korea; 6 Department of Intelligent Semiconductors, Soongsil University, Seoul, South Korea; 7 Department of Mechanical Engineering and Materials Science, Washington University in St. Louis, St. Louis, MO USA; 8 Institute of Materials Science and Engineering, Washington University in St Louis, St Louis, MO USA; 9 Department of Materials Science and Engineering, Massachusetts Institute of Technology, Cambridge, MA USA

**Keywords:** Electrical and electronic engineering, Materials for devices, Nanoscale materials, Electronic devices

## Abstract

Heterogeneous integration of dissimilar crystalline materials has recently attracted considerable attention due to its potential for high-performance multifunctional electronic and photonic devices. The conventional method for fabricating heterostructures is by heteroepitaxy, in which epitaxy is performed on crystallographically different materials. However, epitaxial limitations in monolithic growth of dissimilar materials prevent implementation of high quality heterostructures, such as complex-oxides on conventional semiconductor platforms (Si, III-V and III-N). In this work, we demonstrate gallium nitride (GaN) high-electron-mobility transistors with crystalline complex-oxide material enabled by heterogeneous integration through epitaxial lift-off and direct stacking. We successfully integrate high-κ complex-oxide SrTiO_3_ in freestanding membrane form with GaN heterostructure via a simple transfer process as the gate oxide. The fabricated device shows steep subthreshold swing close to the Boltzmann limit, along with negligible hysteresis and low dynamic on-resistance, indicating very low defect density between the SrTiO_3_ gate oxide and GaN heterostructure. Our results show that heterogeneous integration through direct material stacking is a promising route towards fabricating functional heterostructures not possible by conventional epitaxy.

## Abstract (web summary)

Hyun Kum and colleagues report on the heterogeneous integration of high-k single-crystalline strontium titanium oxide within gallium nitride high-electron-mobility transistors. As the dielectric material is epitaxially grown and transferred, they achieve low defect density between the gate oxide and transistor heterostructures.

## Introduction

Recent advances in producing ultrathin freestanding single-crystalline membranes have enabled heterogenous integration of dissimilar crystalline materials in a single electrical or photonic device, opening a path towards creation of devices with enhanced performance and functionalities^1,2^. The focus of recent studies was mainly on the membrane generation method and a rough demonstration of a prototype device fabricated via heterogeneous integration^3–5^. However, to advance this field to the next stage, it is pivotal to demonstrate the possibility of creating a device with state-of-the-art performance. Many aspects of heterogeneous integration of dissimilar materials are unknown, but perhaps the most important is the interface quality between the transferred single-crystalline membrane and the host heterostructure. To verify this, we have fabricated gallium nitride (GaN)-based high-electron-mobility transistors (HEMT) with a heterogeneously integrated SrTiO_3_ gate oxide layer and characterized its performance. We find that with careful fabrication and transfer of the gate oxide membrane, the device performance in regard to the oxide/HEMT interface show excellent quality, matching or exceeding that of conventionally deposited amorphous gate oxides as well as *in-situ* grown SiN oxides^6–8^.

GaN-based HEMT is one of the most promising structure for high-power and RF applications owing to its superior properties, such as high electron mobility and high breakdown field^9–11^. To further improve the performance and reliability beyond conventional GaN HEMTs with Schottky metal gate, metal-oxide-semiconductor (MOS)-HEMTs structures have been proposed to suppress gate leakage and passivated the GaN surface from ambient. In this regard, MOS-GaN HEMTs with various dielectric materials such as Al_2_O_3_ and HfO_2_ have been demonstrated^12–14^. In MOS-HEMT, the crystallization and interfacial quality of the gate dielectric material play a crucial role in determining the performance^15^.

Among various dielectric materials, complex-oxide materials have attracted considerable interest due to their diverse functional properties such as high dielectric constant (high-κ), ferroelectricity, magnetism, and superconducting properties making complex-oxides an attractive material system for developing next-generation devices^16,17^. However, epitaxial limitations in monolithic growth of dissimilar materials make it difficult to integrate single-crystalline complex-oxide materials with other material platforms such as GaN. The epitaxy of complex-oxides on substrates with different lattice constants and thermal expansion coefficients results in growth of polycrystalline films with inferior material properties. In other words, epitaxial limitations make it difficult to integrate complex-oxide materials onto conventional semiconductors while maintaining its excellent functional properties^18^.

To address this challenge, recent advances in fabricating single-crystalline freestanding complex-oxide membrane techniques have paved the way to seamlessly integrate complex-oxides with any arbitrary semiconductor platforms^19–21^. In this work, we demonstrate state-of-the-art GaN HEMTs utilizing heterogeneously integrated single-crystalline strontium titanium oxide (SrTiO_3_, abbreviated as STO) gate dielectric films. Epitaxial lift-off and direct stacking of freestanding membrane enables integration of crystalline complex-oxide materials on GaN HEMT platforms. STO, a representative perovskite material, exhibits ultrahigh dielectric constant (*κ* ~  300 at room temperature) and comparable breakdown field with conventional dielectric materials^22,23^. This material was recently utilized to demonstrate 2D-FETs successfully^24^. The fabricated devices show excellent electrical characteristics such as negligible hysteresis (Δ*V*), low subthreshold swing (*SS*) close to the Boltzmann limit, and low dynamic on-resistance. We conclude that the pristine interface between the transferred complex-oxide membrane and GaN is attributed to the superior performance of the devices.

## Results

### Structure of the STO/GaN HEMTs

**Fig. 1:**
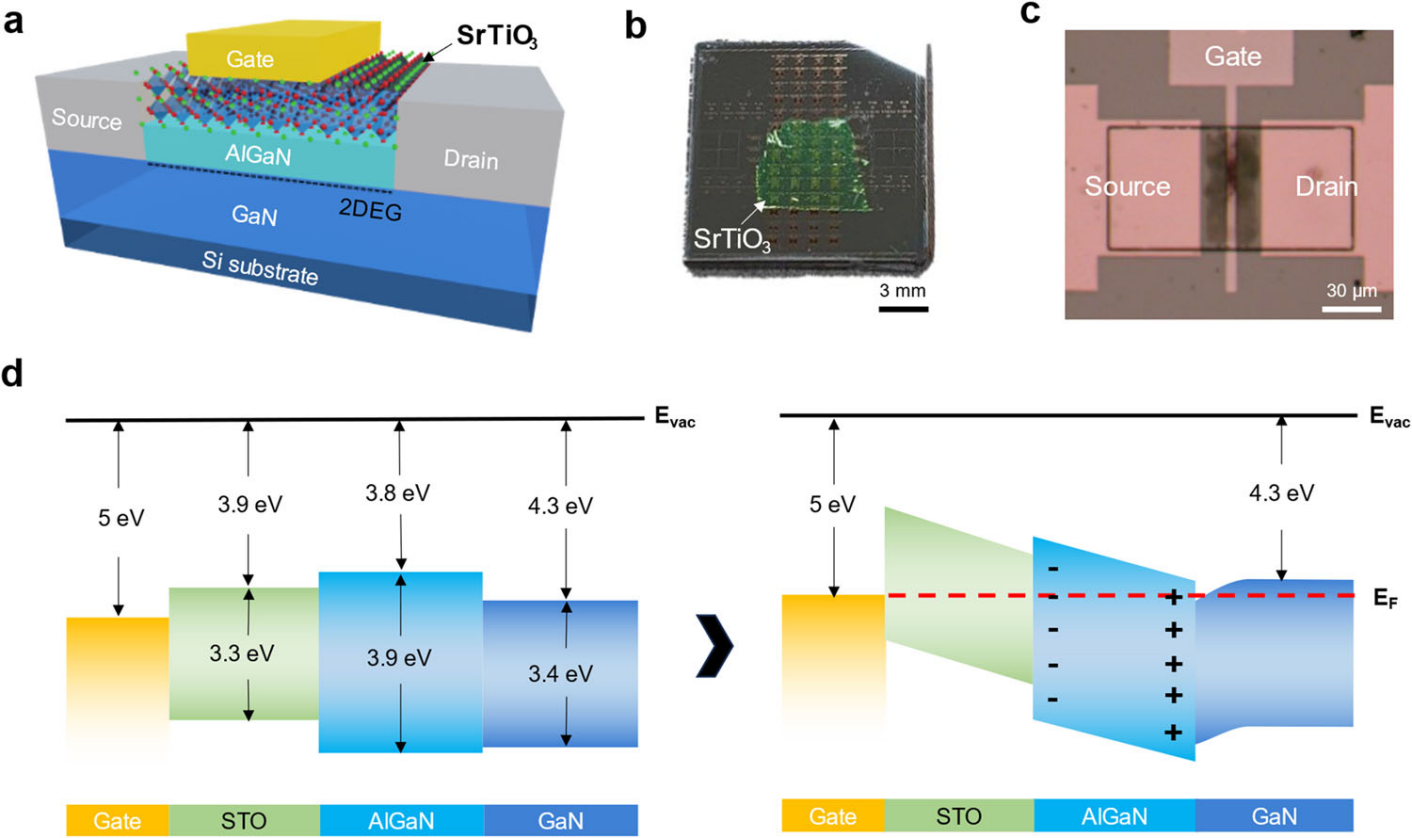
Structure of the STO/GaN HEMTs. **a** Schematic illustration, **b** photograph, **c** optical microscopy image and **d** energy band diagram of fabricated AlGaN/GaN high-electron-mobility transistors (HEMT) with SrTiO_3_ (STO) gate dielectric.

Figure 1a–c shows a 3D schematic illustration, photograph, and optical microscope image of the fabricated STO/GaN HEMT device. Centimeter-scale freestanding STO membrane with a thickness of ~25 nm was transferred onto the AlGaN/GaN HEMT heterostructure as the gate insulator. The channel width, channel length, and gate length are 60 µm, 30 µm, and 4 µm, respectively. Figure 1d shows the energy band diagram of the fabricated device (the characterization and fabrication procedure are shown in Supplementary Figs. 1, 2, respectively). With an estimated valence band offset of ~0.5 eV and conduction band offset of ~0.1 eV, the STO gate insulator forms a type-I straddling band alignment with AlGaN^25–31^. Typically, the type-I negative band alignment of gate dielectric and AlGaN can potentially lead to injection of electrons from the gate toward the interface of the gate insulator and AlGaN due to the low energy barrier to electrons of negative band offset^32^. Furthermore, the injected electrons may be readily captured by trap states near the interface (interface and border traps) that originate from the poor interface quality and defects in the gate insulator. These electron trapping processes may negatively impact the performance of the HEMT device by causing reliability issues, such as decrease in current level and an increase in dynamic on-resistance as a result of decreasing electron density in the 2DEG at the subsequent on-state after the off-state bias stress^33^. However, a clean interface quality and high crystallinity of the gate insulator results in low trap states near the interface, which can compensate for the adverse effects of the type-I negative band alignment and enable highly reliable operation of the devices even with type-I negative band alignment.

### Electrical characteristics

**Fig. 2:**
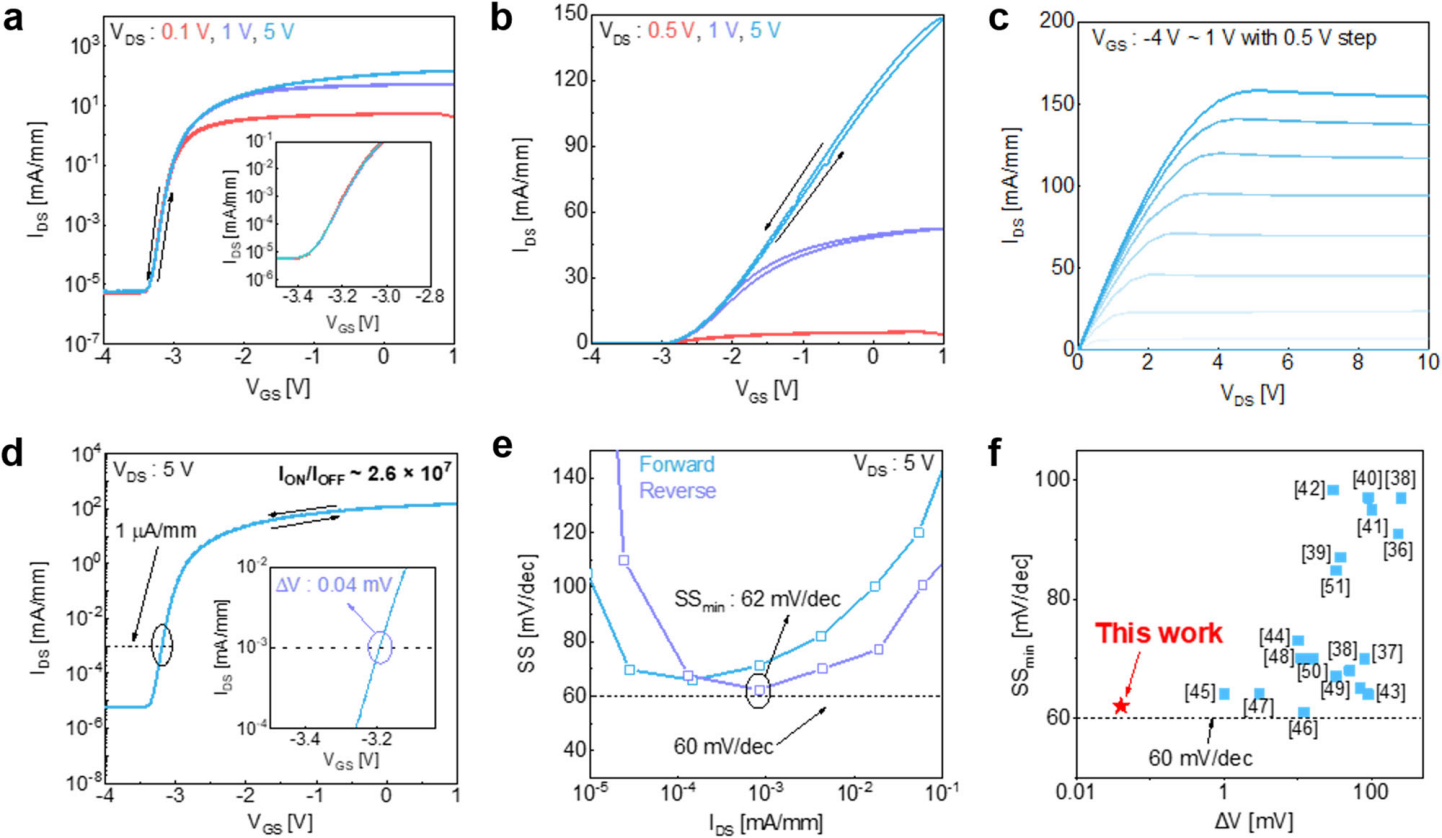
Electrical characteristics. **a** Normalized bidirectional transfer curves of the device at drain-source voltage (*V*_DS_) = 0.1, 1 V, 5 V, respectively in log scale and **b** in linear scale. **c** Normalized output curves in gate-source voltage (*V*_GS_) range of – 4 to 1 V. **d** Normalized bidirectional transfer curves at *V*_DS_ = 5 V. The inset shows hysteresis (*ΔV)* at *I*_DS_ = 1 *µ*A mm^−1^. **e** The subthreshold swing (*SS)* versus I_DS_ curve for forward and reverse direction sweep. **f** Benchmark of minimum *SS* (*SS*_min_) and *ΔV* of the SrTiO_3_ (STO)/GaN high electron mobility transistors (HEMT) compared to previously reported works of metal-oxide-semiconductor (MOS)-HEMTs.

Figure 2 shows the electrical characterizations of the fabricated device. Figure 2a shows the bidirectional transfer characteristics (*I*_DS_-*V*_GS_) normalized with channel width for a drain voltage (*V*_DS_) of 0.1, 1 and 5 V. The inset shows a zoomed in subthreshold region of the transfer curve, showing negligible drain induced barrier lowering (DIBL) for increasing *V*_DS_. Figure 2b shows an identical curve in linear scale, confirming stable device operation with negligible hysteresis, which imply very low defects at the oxide/HEMT interface. To confirm the output characteristics, we measured the output curve (*I*_DS_-*V*_DS_) normalized to the channel width for a *V*_GS_ range from −4 to 1 V, as shown in Fig. 2c. Regarding the pulse operation of digital circuit systems, a large hysteresis during the on-and off switching of the transistor critically degrades the stability and power efficiency of the circuit. Therefore, demonstrating a device with negligible hysteresis is crucial for ensuring stability and increasing power efficiency in digital circuits.

As shown in the Fig. 2d, the device exhibits the ON/OFF ratio ( ~ 2.6 × 10^7^) at *V*_DS_ = 5 V and the hysteresis (Δ*V*) of the device is nearly zero ( ~ 0.04 mA) at 1 *µ*A mm^−1^. Since the hysteresis behavior in MOS-HEMT structures is typically caused by the trapping and detrapping mechanism due to border traps in the gate dielectric^34^, negligible hysteresis of the STO/GaN MOS-HEMT implies that the STO/GaN HEMT is free from border traps owing to the single-crystalline nature of the STO membrane and a clean STO/GaN interface. Furthermore, Fig. 2e shows the subthreshold swing (*SS*) extracted from the bidirectional transfer characteristics, showing an ideally low minimum *SS* (*SS*_min_) value of approximately 62 mV dec^−1^ in both forward and reverse direction, which is close to the Boltzmann limit at room temperature (60 mV dec^−1^). By utilizing the following equation, the interface trap density (*D*_it_) can be estimated from the *SS* value:

1 $${D}_{{{{{{\rm{it}}}}}}}=\frac{{C}_{{{{{{\rm{STO}}}}}}}}{q}\left(\frac{q\,{SS}}{{kT}\;{{{{\mathrm{ln}}}}}10}-1\right)$$

where *k* is Boltzmann constant, *T* is temperature and *C*_STO_ is the capacitance of STO^23,24^. The estimated *D*_it_ at room temperature is approximately 6.33 × 10^11 ^eV^−1^ cm^−2^, which is even lower than MOS-HEMTs with conventional amorphous-based gate dielectric materials^13,35^. Thus, the steep *SS* of our device, which originates from the clean interface of the STO/GaN HEMT, indicates excellent gate controllability, and shows that it’s an advantageous structure for low power consumption applications. Consequently, owing to the pristine interface of the STO/GaN and the high crystallinity of STO, the STO/GaN HEMT exhibits outstanding characteristics in terms of hysteresis and *SS*_min_ compared to the other works, as shown in Fig. 2f^36–51^.

The STO/GaN HEMT shows the high off-state breakdown voltage (*V*_BR_) of 414 V as shown in Supplementary Fig. 3. The breakdown field (*E*_BR_) of device is calculated to be ~2 MV cm^−1^. Supplementary Fig. 4 shows the gate leakage characteristics of the STO/GaN HEMT. It should be noted that the gate leakage in forward bias is considerably affected by the band alignment and barrier height. As shown in Fig. 1d, STO gate insulator forms a type-I straddling band alignment with AlGaN, resulting in higher gate leakage in forward bias region due to the low energy barrier to electrons by negative band offset.

### Dynamic on-resistance degradation

**Fig. 3:**
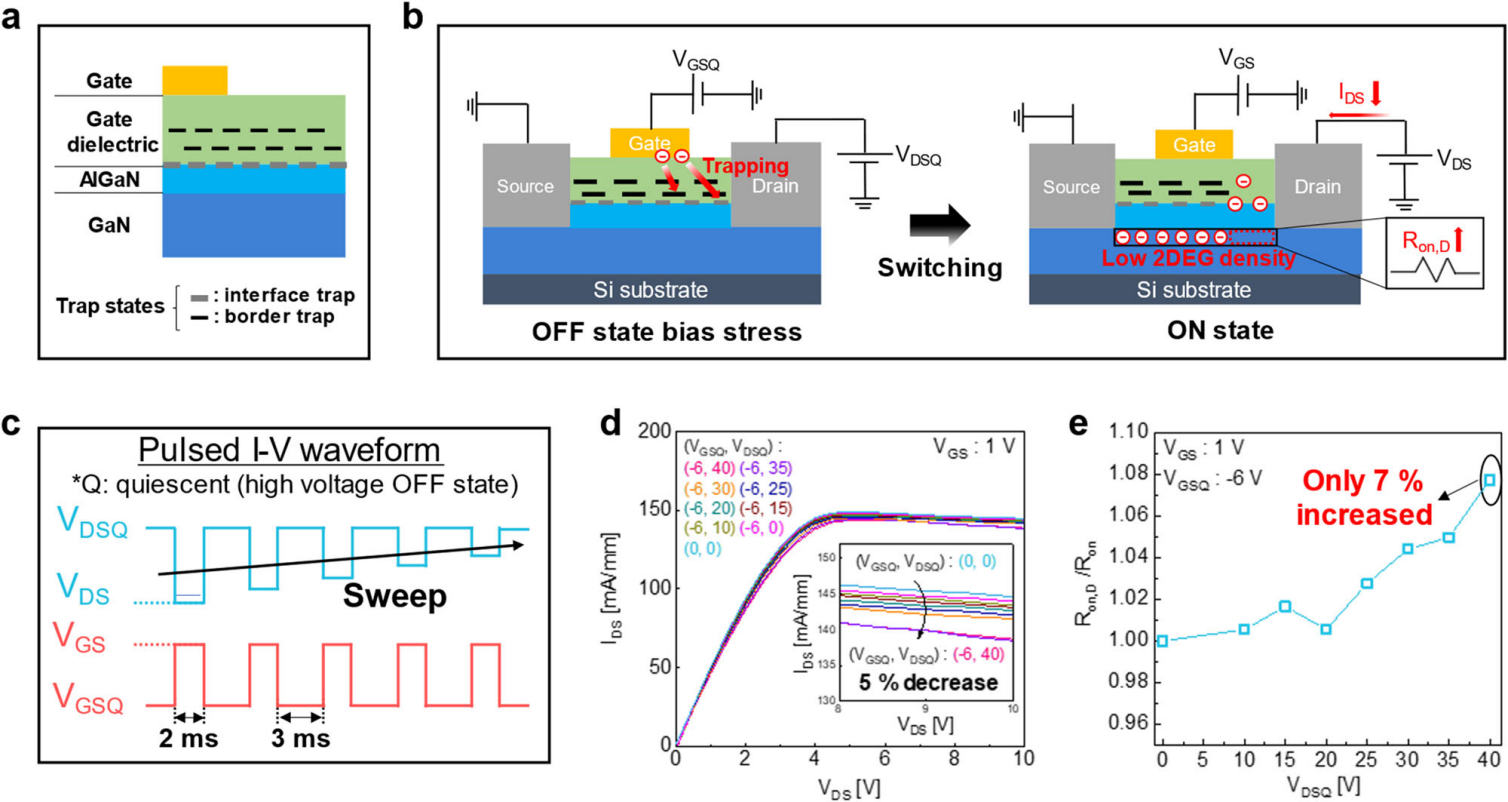
Dynamic on-resistance degradation. **a** Schematic illustration of trap states near the interface. **b** Schematic illustration of physical mechanism of the dynamic on-resistance (*R*_on,D_), as a result of decreased electron density in the two-dimensional electron gas (2DEG) at the subsequent on-state after the off-state bias stress. **c** Synchronous pulse signal of gate-source voltage (*V*_GS_) and drain-source voltage (*V*_DS_) scheme for *R*_on,D_ measurement. **d** Normalized output curve measured with pulsed I-V system for the quiescent drain-source voltage (*V*_DSQ_) range of 0 to 40 V, under the quiescent gate-source voltage (V_GSQ_) of – 6 V. The inset shows only 5% degradation of the on-state current level compared to the initial state during the bias stress. **e** *R*_on, D_/*R*_on_ ratio as a function of *V*_DSQ_.

To further investigate the interface quality of our STO/GaN HEMT device, we measured the dynamic on-resistance (*R*_on,D_) by varying the quasi-drain voltage (*V*_DSQ,_), which indicate the reliability of HEMTs under high quasi-bias stress. Figure 3a, b illustrates the trap states near the interface (interface and border traps) and the mechanism of the dynamic on-resistance effect, respectively. The *R*_on,D_ indicates the ratio of *R*_on_ before and after applying a stress voltage, *V*_DSQ_, at the off state. Due to the trapping of electrons at the interface between the GaN and gate dielectric layer (interface and border traps) during the bias stress, the current degrades after the bias stress, thereby increasing the *R*_on_^52^. While applying a high *V*_DSQ_ at the off state (*V*_GS_ < *V*_TH_), the potential differences between the gate and drain electrode generate the electric field in the vertical direction, inducing electrons injection from the gate toward the interface of the gate insulator and AlGaN as shown in Fig. 3b (left). The injected electrons from the gate may be readily captured by trap states near the interface of the GaN and gate dielectric layer that originate from the poor interface quality and defects in the gate insulator, collapsing the current level due to the decreasing electron density in the 2DEG during the on state (*V*_GS_ > *V*_TH_) as shown in Fig. 3b (right). Accordingly, this allows us to investigate the interface quality of the STO/GaN by analyzing the degradation of on-state current level and *R*_on,D_^36,53^. The periodic drain voltage for high quiescent (*V*_DSQ_, *V*_GSQ_) and ON state was applied using a pulsed I-V system. The pulse width of *V*_DSQ_, *V*_GSQ_, and ON state are 3 ms and 2 ms, respectively. The *V*_GSQ_ was kept at −6 V, while *V*_DSQ_ was varied from 0 to 40 V, and the ON state drain voltage swept from 0 to 10 V as shown in Fig. 3c. Figure 3d exhibits the output characteristics for various *V*_DSQ_ and *V*_GSQ_ combinations, showing only 5 % degradation of the on-state current level compared to the initial state (*V*_GSQ_ = *V*_DSQ_ = 0 V) during the bias stress. As the amplitude of *V*_DSQ_ increases, the electric field generated by the potential drops between the gate and drain electrodes is enhanced, leading to the increases of the electron trapping into the interface trap at the GaN/dielectric layer.

To evaluate the influence of *R*_on,D_ as a function of the quiescent bias (*V*_DSQ_), we calculated the *R*_on,D_ by increasing the *V*_DSQ_ from 0 V to 40 V with 5 V steps as shown in Fig. 3e. Owing to the high crystalline quality of STO and the clean interface of the STO/GaN, the maximum *R*_on,D_ only increases up to 7 %, which is much lower compared to conventional GaN MOS-HEMTs utilizing deposited amorphous oxides^54^.

### TEM characterization of STO/GaN interface

**Fig. 4:**
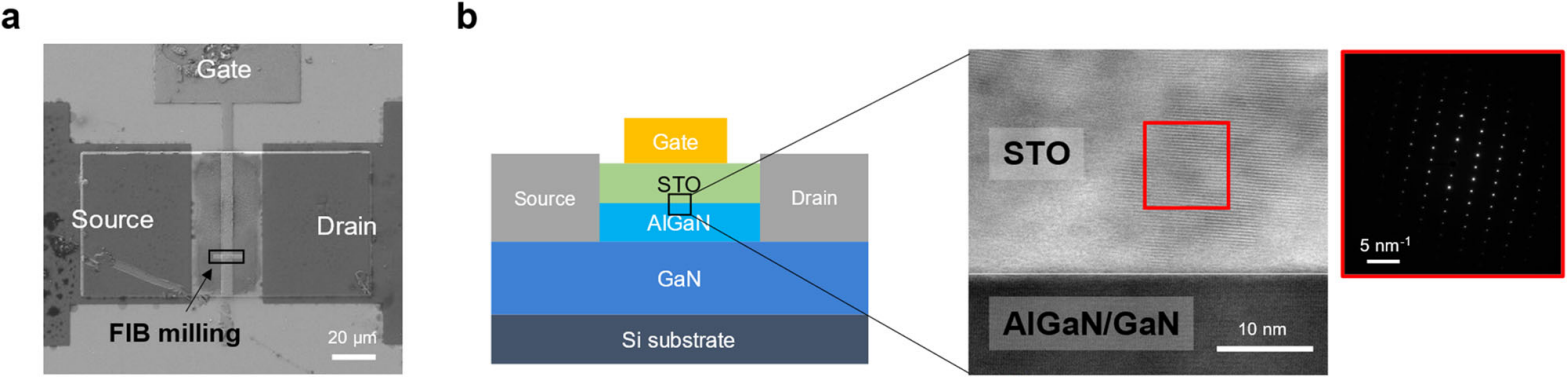
TEM characterization of STO/GaN interface. **a** Plan-view scanning electron microscope (SEM) image of HEMT device, black box represents region of focused ion beam (FIB) milling for transmission electron microscope (TEM) analysis. **b** Cross-sectional TEM image of SrTiO_3_ (STO)/GaN interface. The inset shows selected area electron diffraction (SAED) pattern of the transferred STO.

Finally, to directly investigate the interface of the STO/GaN HEMT, a cross-sectional TEM measurement was conducted. Figure 4a shows the top view SEM image of the fabricated STO/GaN HEMT device and the investigated area of the TEM sample. The TEM was performed at the AlGaN/STO interface, (including a GaN capping layer) as illustrated in the left image of Fig. 4b. Figure 4b (right image) shows the TEM image of at the STO/GaN/AlGaN interface, which showed no defects, airgaps, unexpected interfacial layers or residues at the STO/GaN interface. We suspect that the dangling bonds of the transferred STO membrane may form atomic bonding with the underlying substrate (in our case, GaN HEMT heterostructure) by the thermal annealing process, which consistent with the previous works^55–59^ (the mechanism of the interface bonds formation is schematically illustrated in Supplementary Fig. 5). Moreover, the selected area electron diffraction (SAED) pattern (inset) of the STO region, verified the crystalline nature of the transferred STO membrane. We believe these results, along with the electrical analysis of the gate oxide interface, strongly support that the reliable operation and excellent performance of the fabricated STO gate oxide HEMT is a result of a pristine interface between highly crystalline STO gate oxide and GaN.

## Conclusions

In summary, we heterogeneously integrated crystalline complex-oxide material on AlGaN/GaN HEMT as a gate dielectric by epitaxial layer transfer approach. Using STO with ultrahigh-κ properties as the gate oxide, we fabricated a MOS-HEMT device. The fabricated devices exhibited a negligible hysteresis (Δ*V*) at a drain current of 1 µA/mm, and a minimum *SS* value of 62 mV dec^−1^. We attribute these results to an extremely clean interface between the STO and GaN, free from interface and border traps. The dynamic on resistance measurements were performed for further interface quality analysis, in which our device only showed a maximum resistance increase of 7%.

Finally, we confirmed through TEM that no unwanted interfacial layer, residues, or airgaps between STO/GaN exists, and that the transferred STO maintains its crystalline nature. Our results demonstrate the potential of heterogeneous integration of complex-oxide materials with mature semiconductor technologies, substantially expanding the possibility of creating high performance electrical and photonic devices with novel functionalities.

## Methods

### Material preparation and device fabrication

A GaN HEMT, consisting of GaN capping layer (3 nm), Al_0.26_Ga_0.74_N barrier (25 nm), AlN interlayer (1 nm), GaN channel layer (2 *μ*m) and GaN buffer layer (1 *μ*m), was grown via metal-organic chemical vapor deposition (MOCVD) on a Si (111) substrate. The electron mobility and sheet carrier concentrations of the two-dimensional electron gas (2DEG) formed between the AlGaN/GaN interface were >1300 cm^2^ V^−1^ s^−1^ and ~10^13 ^cm^−2^ at *T* = 300 K.

The integration of STO with AlGaN/GaN HEMT structure begins with the successive epitaxial growth of a water-soluble strontium aluminum oxide (Sr_3_Al_2_O_6_, abbreviated as SAO) sacrificial layer, followed by the growth of STO gate oxide on a single-crystalline STO (001) substrate by pulsed-laser deposition (PLD). It has been reported that the epitaxial growth of oxide film on the SAO template allows the transfer of single-crystalline STO membranes without fundamental thickness limitation^60^. The single-crystallinity of the epitaxially grown STO film on SAO/STO substrate is confirmed using X-ray diffraction (XRD) and electron backscatter diffraction (EBSD) map, as shown in Supplementary Fig. 1a–c. After deposition of a poly(methyl methacrylate) (PMMA) supporting layer on the as-grown STO/SAO/STO substrate via spin-coating, the stack was immersed in deionized (DI) water for ~24 hours to completely dissolve the SAO sacrificial layer^61^. The freestanding STO membrane with a thickness of ~25 nm was then transferred onto the AlGaN/GaN HEMT structure followed by removal of the supporting layer by acetone and rinsed by isopropyl alcohol (the complex-oxide membrane transfer procedure schematically illustrated in Supplementary Fig. 2).

The transferred membrane was patterned through standard photolithography and etched using a combination of ion milling and etching in diluted hydrofluoric acid (HF) solution. We believe that the HF etch removes most of the defects caused by the ion milling in the gate oxide membrane, leading to excellent characteristics. Mesa isolation was conducted by inductively coupled plasma reactive ion etching (ICP-RIE) using a mixture of BCl_3_/Cl_2_ gas. A Ti/Al/Ti/W (20/120/20/30 nm) stack was deposited for the source/drain electrode via e-beam evaporation, followed by rapid thermal annealing at 500 °C for 2 min in N_2_ environment. Finally, a Ni film ( ~ 500 nm) was deposited as the gate electrode via plasma sputtering (the low magnification cross-sectional image of the fabricated device is shown in Supplementary Fig. 6).

### Electrical and material characterizations

The electrical properties of the fabricated devices were measured using a semiconductor parameter analyzer (Keithley−4200A-SCS) with a 4255-RPM module to apply pulsed signals. The structural and interfacial analysis was performed using a scanning electron microscope (SEM), focused ion beam (FIB), and transmission electron microscopy (TEM). SEM measurements were performed using JEOL high-resolution SEM (IT-500HR), ZEISS SEM with an EBSD detector. The cross-sectional TEM specimens of the fabricated devices were prepared using a Ga-focused ion beam milling (ZEISS crossbeam 540) technique. TEM measurements were performed using a JEOL ARM 200 F (NEOARM).

### Supplementary information

Supplementary information

## Data Availability

The data that support the findings of this study are available from the corresponding author upon reasonable request.
