# Supplementary material for: Heterogeneous integration of high-k complex-oxide gate dielectrics on wide band-gap high-electron-mobility transistors
Source: Commun Eng. 2024 Jan 19;3:15. doi: 10.1038/s44172-024-00161-z (PMC10955896; doi:10.1038/s44172-024-00161-z)
Supplement: Supplementary file 1 — Supplementary information [file 44172_2024_161_MOESM1_ESM.pdf]

Supplementary information

Heterogeneous integration of high-k complex-oxide gate dielectrics on wide band-gap high-electron-mobility transistors

Jongho Ji[1†], Jeong Yong Yang[2†], Sangho Lee[3,4†], Seokgi Kim[5], Min Jae Yeom[2], Gyuhyung Lee[2,6], Heechang Shin[1], Sang-Hoon Bae[7,8], Jong-Hyun Ahn[1], Sungkyu Kim[5], Jeehwan Kim[3,4,9*], Geonwook Yoo[2,6*], Hyun S. Kum[1*]

[1]Department of Electrical and Electronic Engineering, Yonsei University, Seoul, South Korea
[2]Department of Electronic Engineering, Soongsil University, Seoul, South Korea.
[3]Department of Mechanical Engineering, Massachusetts Institute of Technology, Cambridge, MA USA
[4]Research Laboratory of Electronics, Massachusetts Institute of Technology, Cambridge, MA USA
[5]Department of Nanotechnology and Advanced Materials Engineering, Sejong University, Seoul, South Korea
[6]Department of Intelligent Semiconductors, Soongsil University, Seoul, South Korea.
[7]Department of Mechanical Engineering and Materials Science, Washington University in St. Louis, St. Louis, MO, USA
[8]Institute of Materials Science and Engineering, Washington University in St Louis, St Louis, MO, USA
[9]Department of Materials Science and Engineering, Massachusetts Institute of Technology, Cambridge, MA USA

* Indicates corresponding authors
† These authors contributed equally: Jongho Ji, Jeong Yong Yang, Sangho Lee

* Corresponding author: Jeehwan Kim, Geonwook Yoo, Hyun S. Kum
* E-mail address: jeehwan@mit.edu, gwyoo@ssu.ac.kr, hkum@yonsei.ac.kr

Supplementary Fig. 1

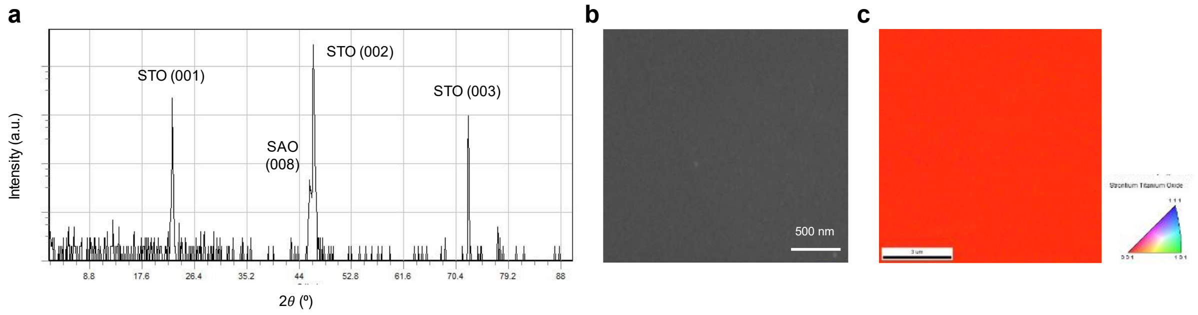

Supplementary Fig. 1 | (a) XRD of epitaxially grown STO/SAO film on a STO (001) substrate. Plan-view (b) SEM and (c) EBSD mapping image of STO film.

Supplementary Fig. 2

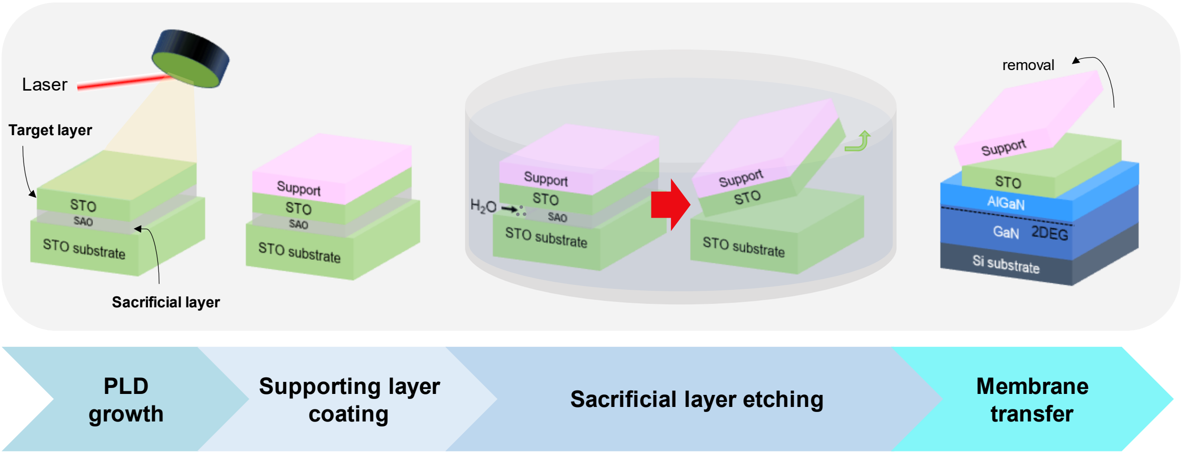

Supplementary Fig. 2 | Schematic illustration of complex-oxide membrane transfer process

Supplementary Fig. 3

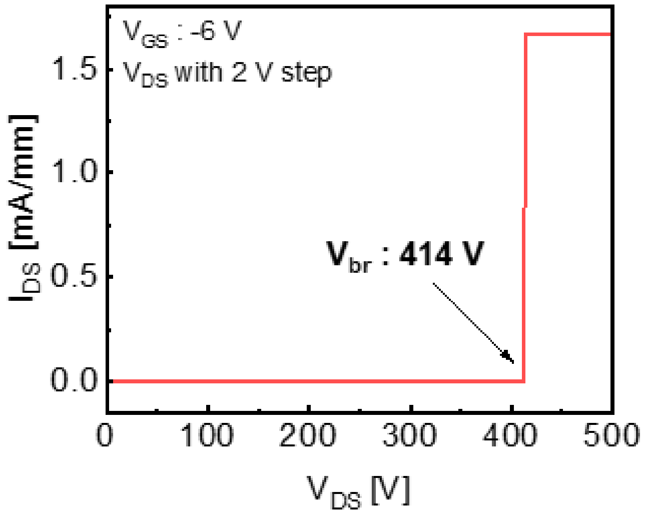

Supplementary Fig. 3 | OFF-state breakdown characteristics (I_{DS}-V_{DS}) of the device at V_{GS} = -6 V (L_{GD} = 2.3 µm).

Supplementary Fig. 4

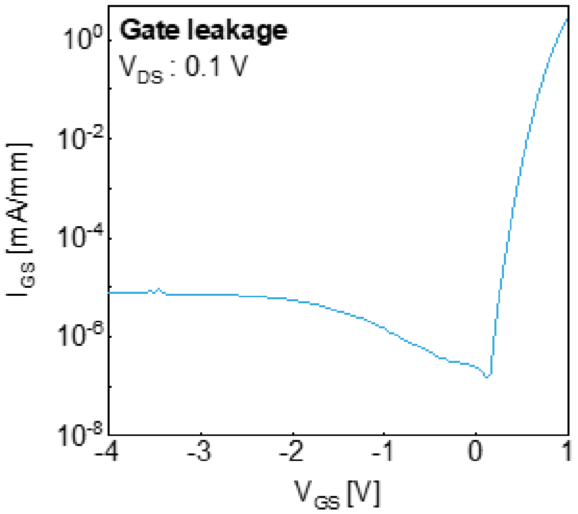

Supplementary Fig. 4 | Gate current characteristics (I_{GS}-V_{GS}) of the device at V_{DS} = 0.1 V.

Supplementary Fig. 5

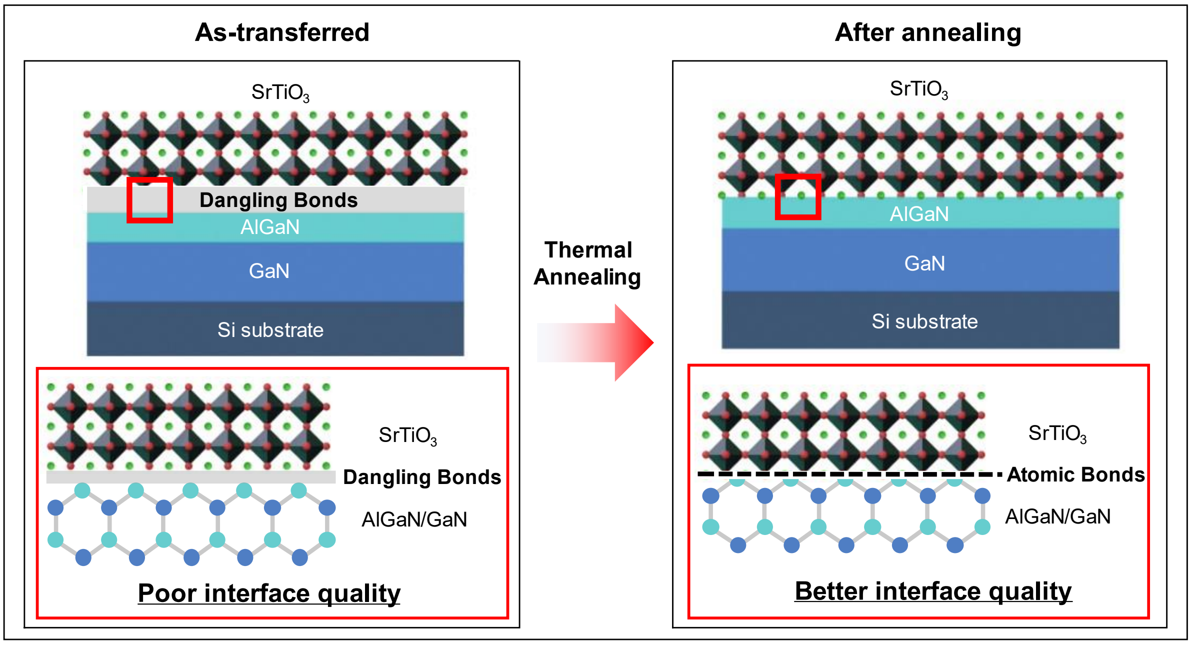

Supplementary Fig. 5 | Schematic illustration of the possible mechanism of the bonds formation at the interface.

Supplementary Fig. 6

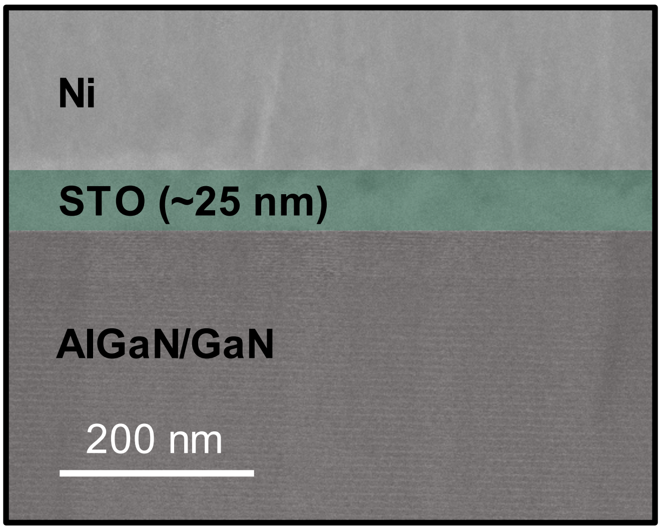

Supplementary Fig. 6 | Low-magnification cross-sectional TEM image of the STO/GaN HEMT.
